# Percutaneous Transhepatic Venous Embolization and Portal Vein Stenting for Ectopic Variceal Bleeding at Choledochojejunostomy After Pancreaticoduodenectomy With Portal Vein Stenosis: A Case Report

**DOI:** 10.7759/cureus.75374

**Published:** 2024-12-09

**Authors:** Masanori Odaira, Nobutake Ito, Yuki Iwaita, Kota Tanuma, Hirohisa Harada

**Affiliations:** 1 Department of Surgery, Tokyo Saiseikai Central Hospital, Tokyo, JPN; 2 Department of Radiology, Tokyo Saiseikai Central Hospital, Tokyo, JPN; 3 Department of Gastroenterology, Tokyo Saiseikai Central Hospital, Tokyo, JPN

**Keywords:** choledochojejunostomy, ectopic varices, pancreaticoduodenectomy, portal vein stenosis, portal vein stent, variceal embolization

## Abstract

Ectopic varices can result from portal vein stenosis following pancreaticoduodenectomy with concomitant portal vein resection reconstruction, and they can cause gastrointestinal bleeding. Although they can sometimes be fatal, various treatments have been reported. This report describes a case in which a percutaneous transhepatic approach was used to simultaneously perform variceal embolization and portal vein stenting in which a favorable outcome was achieved.

The patient was a 77-year-old woman who had undergone subtotal stomach-preserving pancreaticoduodenectomy and portal vein combined resection and reconstruction for stage IIA pancreatic cancer. Although postoperative portal vein stenosis was observed, the patient was followed up because the collateral blood flow was well developed, maintaining intrahepatic blood flow. About 18 months after surgery, the day before a routine outpatient visit, she noticed melena, and a blood test performed at the time of the outpatient visit revealed anemia. An emergency contrast-enhanced computed tomography and an emergency enteroscopy revealed ectopic varices around the elevated jejunum at the choledochojejunostomy, and bleeding from the same site was suspected. Since the patient was suspected to be suffering from portal hypertension, we planned to embolize the varices for bleeding and to place a portal stent to treat portal hypertension. Since the patient had undergone mesh placement for an incisional hernia approximately one year postoperatively, a percutaneous transhepatic route was selected, and the patient was approached via the right portal vein route. The varices were embolized with coils and histoacrylate, and a stent was placed in the stenotic portal vein. The portal vein pressure was measured before and after the procedure to confirm its reduction, and the procedure was completed without complications. The patient was discharged from the hospital seven days after the procedure with no problems and is currently under outpatient follow-up with no varice recurrence.

Although the optimal treatment for ectopic varices has not been established, portal vein stenting and variceal embolization via a percutaneous transhepatic approach were effective.

## Introduction

Portal vein (PV) stenosis can occur following tumor invasion of the hilar or pancreatic head region or postoperative inflammation in the same area [1-3]. It may also be common in patients with concomitant PV resection who have undergone reconstructive surgery. PV stenosis can cause portal hypertension and lead to the development of ectopic varices as collateral vasculature, especially in post-pancreatoduodenectomy patients, can result in varices at the choledochojejunostomy, and it is critical to diagnose and treat such varices because they can cause sudden gastrointestinal bleeding and are sometimes fatal. Sometimes, diagnosing ectopic varices is relatively easy via computed tomography (CT); however, in other cases, it may require small bowel endoscopy or angiography. Various treatment methods have been reported, including methods for the treatment of the ectopic varices themselves and for portal hypertension. Endoscopic treatment [4-6], interventional radiology (IVR) [7-14], and surgery [15] have been reported for both conditions; however, no validated therapy exists yet. Herein, the current report describes a case of ectopic varices at the choledochojejunostomy caused by PV stenosis and portal hypertension after pancreaticoduodenectomy with PV resection and reconstruction for pancreatic cancer, which was successfully treated by embolization of the varices and portal stent placement via a percutaneous transhepatic approach.

## Case presentation

**Figure 1:**
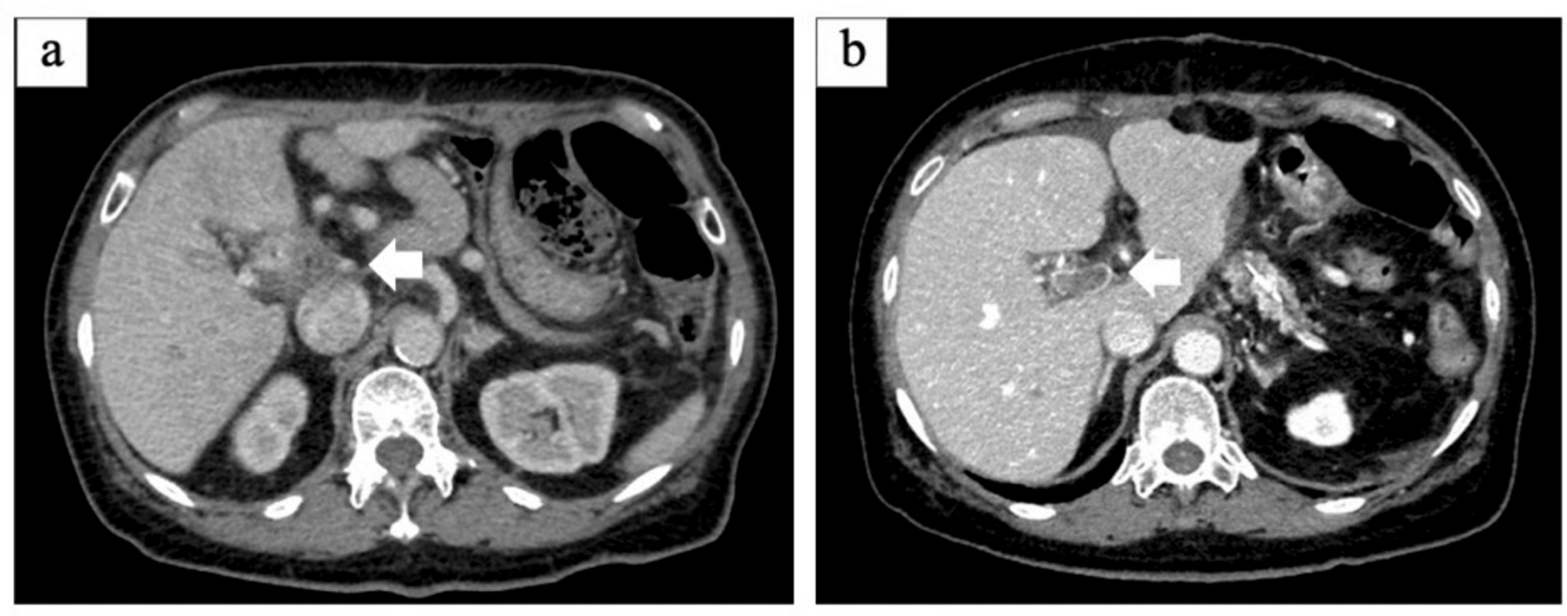
Postoperative one-week axial CT images (a) Portal vein stenosis (arrow); (b) Portal vein thrombus (arrow)

The patient was a 77-year-old woman who was diagnosed with stage IIA pancreatic head cancer and scheduled for surgery after two courses of preoperative chemotherapy comprising two courses of S-1 and Gemcitabine. After chemotherapy, she underwent subtotal stomach-preserving pancreaticoduodenectomy (PD) and PV combined resection and reconstruction because the tumor was suspected to have invaded the PV. PV reconstruction was performed with an end-to-end anastomosis and continuous suturing with 5-0 nonabsorbable thread. The surgery duration was 525 min, and the estimated intraoperative blood loss was 915 ml. One week postoperatively, a follow-up CT scan revealed stenosis and a thrombus in the PV (Figures 1a, 1b).

However, thanks to the development of collateral vessels, intrahepatic blood flow was preserved, and the patient was asymptomatic; therefore, she was given a direct oral anticoagulant (DOAC) and followed up strictly. After starting the DOAC, the patient experienced no major problems and was discharged on postoperative day 23. She was followed up on an outpatient basis without major problems or recurrence. Approximately one year postoperatively, a mesh-assisted radical repair for an incisional hernia was performed.

**Figure 2:**
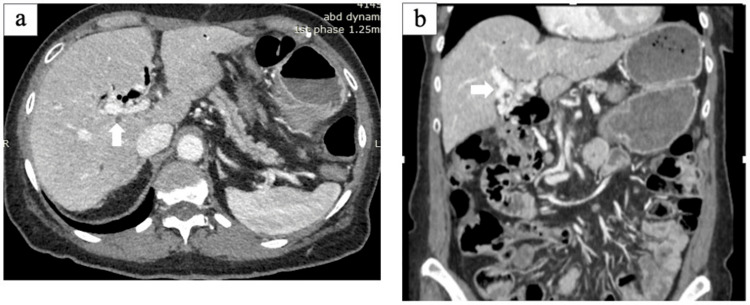
CT performed during gastrointestinal bleeding (a, b) Axial and coronal CT images show ectopic varices around the choledochojejunostomy (arrow)

About 18 months postoperatively, the day before a routine outpatient visit, she noticed melena. During her outpatient visit, she was hemodynamically stable but markedly anemic with a hemoglobin level of 6.0 g/dl (11.7 g/dl 3 months earlier). An emergency contrast-enhanced CT scan revealed ectopic varices around the elevated jejunum at the choledochojejunostomy, and bleeding from the same site was suspected (Figures 2a, 2b).

**Figure 3:**
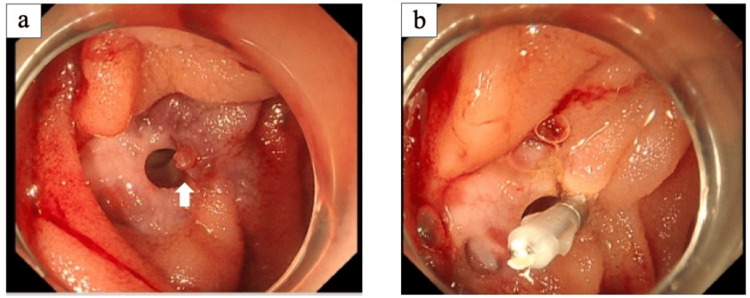
Enteroscopy findings (a) The bleeding point from ectopic varices (arrow); (b) Clipping performed for hemostasis

To identify the origin of the bleeding, an endoscopy was performed. An emergency enteroscopy with a single-balloon scope revealed the same findings as the CT scan but with a confirmed bleeding point. Cauterization and clipping were successfully performed for primary hemostasis (Figures 3a, 3b).

The patient was admitted urgently for close follow-up, and her general condition stabilized with a blood transfusion. The cause of the ectopic varices was determined to be portal hypertension associated with PV stenosis, and radical treatment was considered. IVR sclerotherapy for varices and stenting for PV stenosis via a percutaneous transhepatic route was performed, partly because of the mesh implantation in the abdominal wall.

**Figure 4:**
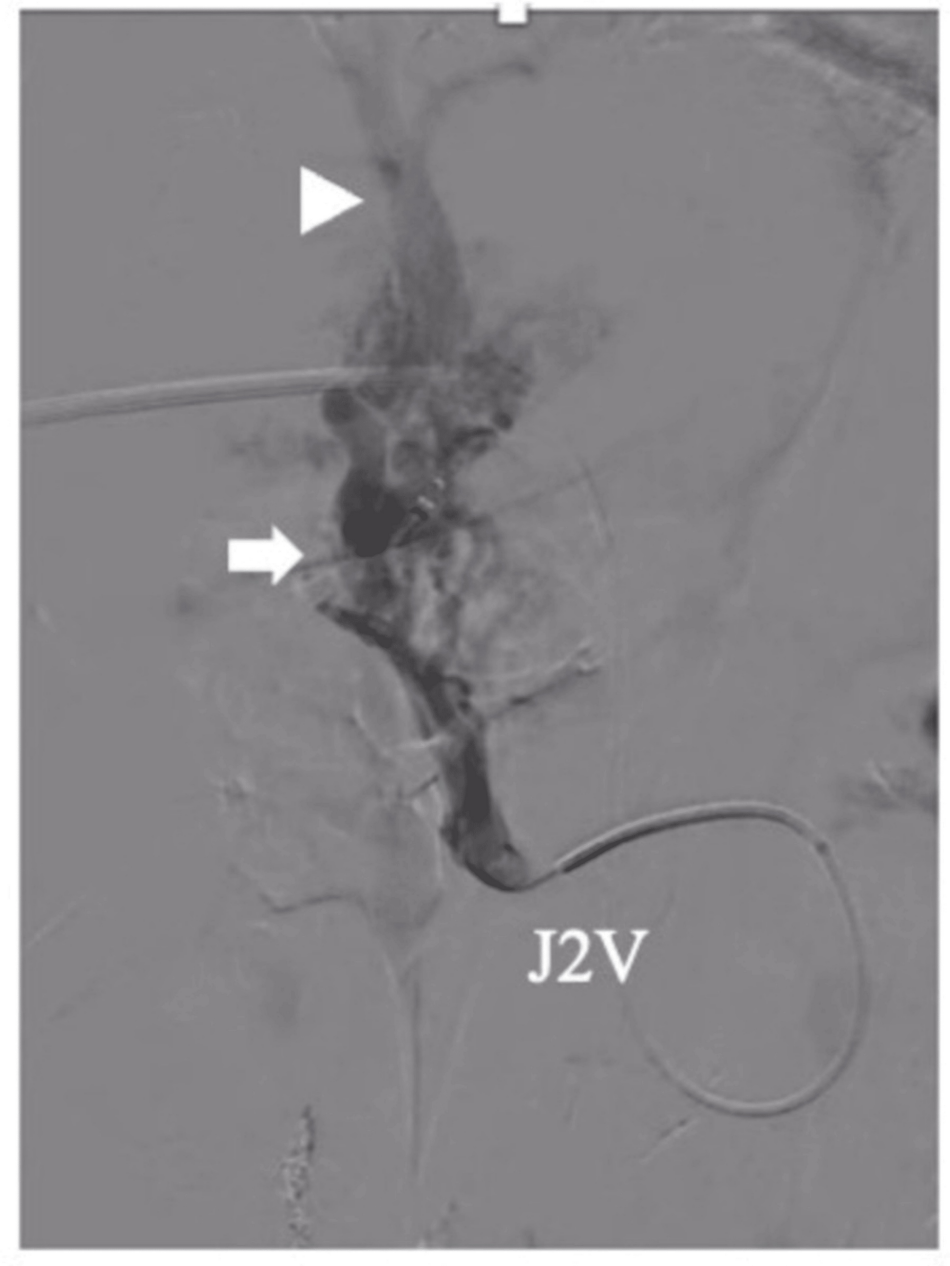
Angiogram from the second jejunal vein Ectopic varices were contrasted (arrow), and they flowed into the intrahepatic portal vein (arrowhead).

The PV’s anterior segment branch was punctured with echo guidance and a catheter was inserted under local anesthesia. Catheter-based pressure measurements revealed a superior mesenteric vein (SMV) pressure of 17 mmHg and PV pressure of 15 mmHg. A catheter was advanced into the second jejunal vein and contrast enhancement was performed, revealing varices and a marked shunt to the PV’s anterior segmental branch (Figure 4).

**Figure 5:**
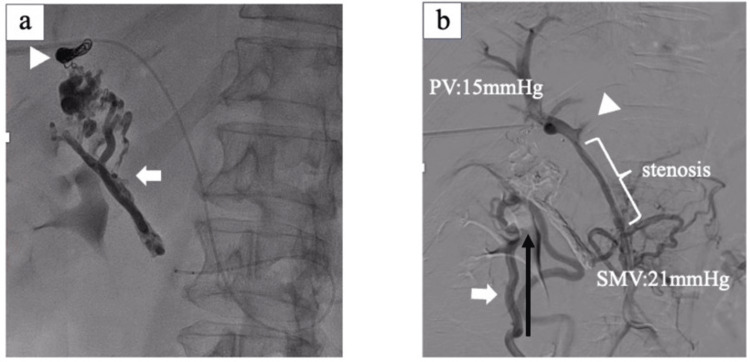
Portography after sclerotherapy of ectopic varices (a) Coil embolization of the variceal outflow tract (arrowhead) and cyanoacrylate variceal sclerotherapy (arrow); (b) Ileocecal vein regurgitation (arrowhead) and poor contrast of the left portal vein (arrowhead)

Coil embolization of the shunt outflow was preceded by prevention for inflow into the liver, followed by sclerotherapy with histoacrylate. Post-treatment contrast enhancement revealed not only adequate embolization of the varices but also regurgitation of the ileocecal vein and left PV branch (Figure 5a, 5b).

**Figure 6:**
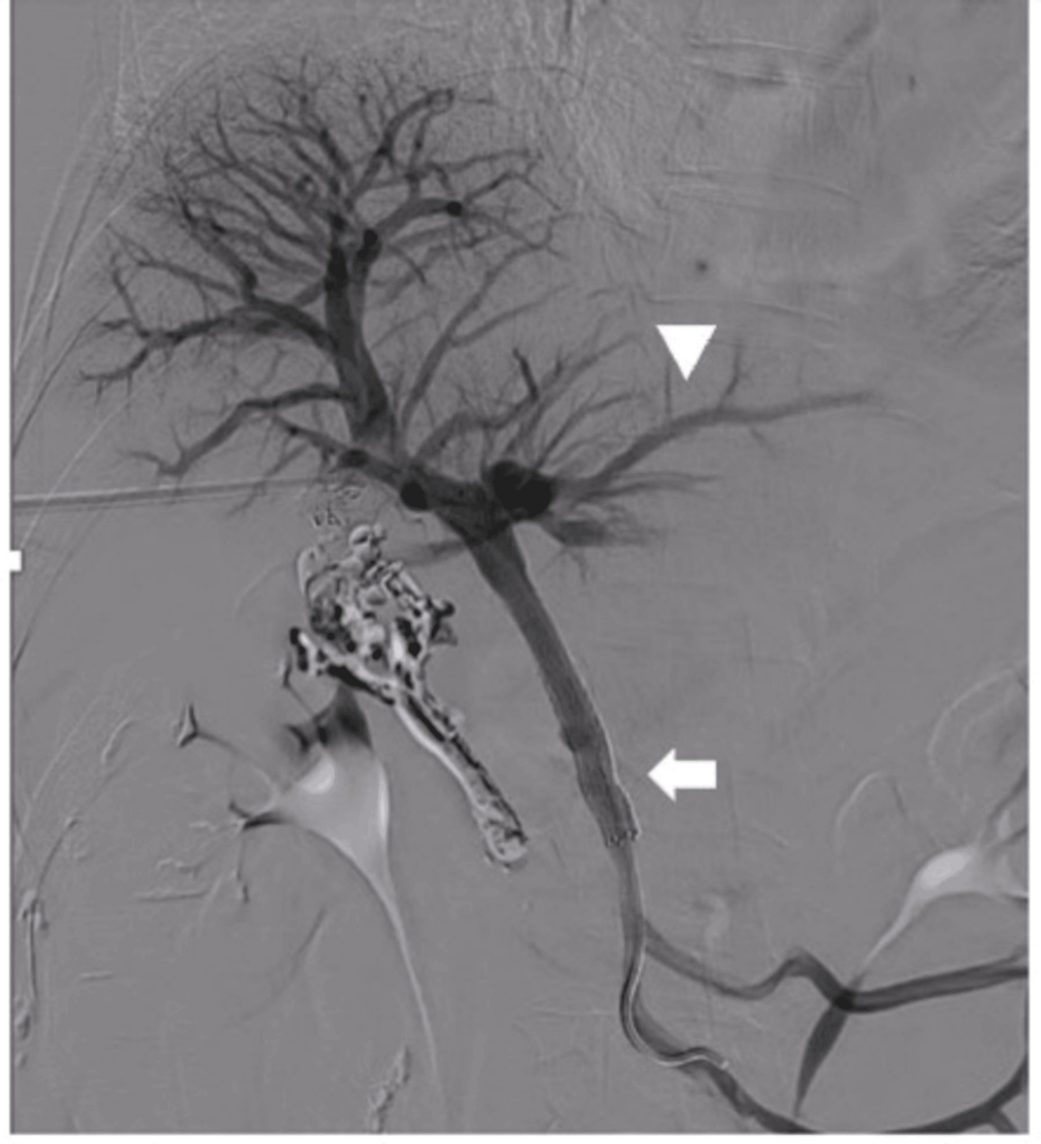
Portography after portal stent insertion Stenosis improvement due to portal vein stent insertion (arrow) and progressive left portal vein contrast (arrowhead)

Again, the pressure of the SMV and PV was measured using a catheter and found values of 21 mmHg and 15 mmHg, respectively, which is considered a significant finding. The treatment plan was to insert a PV stent as scheduled, and an 8 cm self-expandable metallic stent with a 9 mm diameter was inserted into the vessel’s stenotic segment and crimped by balloon dilation. Contrast studies after stent insertion revealed good portal dilation and intrahepatic blood flow, and the left PV was also progressive. Ileocolonic vein regurgitation also disappeared (Figure 6).

Catheter pressure measurements revealed an SMV pressure of 18 mmHg, and a PV pressure of 17 mmHg, respectively, and the pressure differential was improved. The scheduled procedure was successfully performed and terminated. The total time required for IVR was 192 min. The patient’s post-IVR course was stable. A CT scan performed three days after IVR revealed stent patency, hemostasis, and embolization of the varices. After seven days of IVR, she was discharged from the hospital, after which she continued taking DOAC and is under outpatient follow-up. Twenty-one months post-IVR, she is doing well with no PV restenosis or rebleeding.

## Discussion

PD is a procedure for refractory malignancies such as pancreatic cancer and distal bile duct cancer. This highly invasive procedure requires attention to short-term complications such as pancreatic fistulas. However, with recent advances in surgery and chemotherapy, the number of long-term survivors after PD has increased. In addition, the number of cases of low-grade conditions, such as intraductal papillary mucinous neoplasms, is increasing, and so is the number of times the procedure itself is performed. With the increase in the number of long-term survivors, there is also an increased chance of encountering long-term complications such as postoperative cholangitis and diabetes mellitus. PV stenosis is one of the most significant long-term complications post-PD. It is caused by inflammation associated with postoperative pancreatic and biliary fistulas, as well as PV resection and reconstruction. The estimated incidence of portal vein stenosis post-PD is 19.6%, and in approximately half of PV complication cases, resection and reconstruction resulted in stenosis [1]. PV stenosis causes portal hypertension, which can lead to ectopic varices. These varices are particularly known to occur easily at the choledochojejunostomy in post-pancreaticoduodenectomy patients. Ectopic varices sometimes cause gastrointestinal bleeding, which is associated with a mortality rate of up to 40% [16]. Bleeding from varices has been reported in approximately 3% of portal stenosis cases [1]. Because of its anatomical characteristics, there is usually no hematemesis, and the main clinical manifestation is melena, as in the present case. The first step in the diagnosis of this condition is contrast-enhanced CT; however, some cases are difficult to diagnose; therefore, enteroscopy or angiography is useful for a more detailed evaluation of these differences.

The treatment of choice for this condition needs to concomitantly address the varices and portal hypertension. Treatment of varices can be divided into ligation and sclerotherapy. Surgical ligation is less feasible due to its invasive nature, and an endoscopic or IVR approach is often employed, including the identification of the bleeding site. The endoscopic approach is becoming an option without special facilities with the widespread use of enteroscopy. In this case, although the diagnosis by enteroscopy was useful, the patient was not prepared for sclerotherapy; therefore, endoscopic radical treatment was not performed, and temporary hemostasis was achieved by clipping and cauterization. In IVR, the approach of choice is an echo-guided percutaneous transhepatic route or an ileal vein route with an open abdomen. Although the echo-guided approach may be technically challenging, it is minimally invasive and should be the first-choice approach. In both methods, there are many reports of good outcomes with the treatment of varices alone [9,11,14]; however, considering the pathophysiology of the condition, radical treatment for portal hypertension is necessary.

Treatment options resulting from postoperative PV stenosis include shunt surgery and PV stent insertion. Shunt surgery is invasive, and the procedure may be difficult because of postoperative adhesions. Therefore, the number of reported cases of PV stenting for postoperative PV stenosis has increased in recent years. As with IVR for varices, the approach to PV stenting can be divided into an echo-guided percutaneous transhepatic route or an ileocecal vein route with an open abdomen. When echo-guided and performed by a skilled IVR physician, the percutaneous transhepatic route is feasible and appears to be less invasive than an open-abdomen procedure. Portal stent insertion has been reported in many cases with high success rates and low complication rates, and it is considered to be an extremely useful treatment option for PV stenosis [7,8,10-13]. The long-term patency rate of PV stents is reported to be approximately 80% [17]. To the best of our knowledge, there is no unified view on the need for anticoagulants after PV stent insertion, with some cases requiring anticoagulant use and others reporting concerns about bleeding complications [18]. We believe that anticoagulants are effective in preventing obstruction due to thrombus formation, and we have continued their administration in this case after treatment, with no apparent bleeding complications.

In a case of PV stenosis with ectopic varices similar to the present case, there are reports of no treatment for varices because PV stenting provides a therapeutic effect against portal hypertension and the resolution of variceal visualization. However, in a report of long-term outcomes after PV stent placement, only the presence or absence of varicose vein sclerotherapy significantly affected the patency rate [19], and we used this as a reference to embolize the varices. This is thought to be due to a reduction in portal blood flow velocity because varicose vein embolization was not performed. Since portal stent placement and variceal embolization can be performed through the same approach route, sclerotherapy should also be performed if possible.

There are various approaches to treating ectopic varices that coexist with PV stenosis. Although one-stage treatment under IVR (as performed in this case) is ideal, it is most important to select the optimal treatment method in consultation with the surgeon, endoscopist, and interventional radiologist, depending on the facility, the physician’s skills, and the patient’s condition.

## Conclusions

In this study, the report describes a case of ectopic variceal bleeding around the choledochojejunostomy caused by portal vein stenosis after PD. It was successfully treated with endoscopic hemostasis, IVR variceal embolization, and PV stenting. The IVR procedure was considered minimally invasive, therapeutic, and instrumental.
